# Static body postural misalignment in individuals with temporomandibular disorders: a systematic review

**DOI:** 10.1590/bjpt-rbf.2014.0061

**Published:** 2014

**Authors:** Thaís C. Chaves, Aline M. Turci, Carina F. Pinheiro, Letícia M. Sousa, Débora B. Grossi

**Affiliations:** 1 Departamento de Neurosciências e Ciências do Comportamento, Faculdade de Medicina de Ribeirão Preto (FMRP), Universidade de São Paulo (USP), Ribeirão Preto, SP, Brazil; 2 Departamento de Biomecânica, Medicina e Reabilitação do Aparelho Locomotor, FMRP, USP, Ribeirão Preto, SP, Brazil; 3 Departamento de Medicina Social, FMRP, USP, Ribeirão Preto, SP, Brazil

**Keywords:** temporomandibular disorders, body posture, craniocervical posture, systematic review

## Abstract

**BACKGROUND:**

The association between body postural changes and temporomandibular disorders (TMD) has been widely discussed in the literature, however, there is little evidence to support this association.

**OBJECTIVES:**

The aim of the present study was to conduct a systematic review to assess the evidence concerning the association between static body postural misalignment and TMD.

**METHOD:**

A search was conducted in the PubMed/Medline, Embase, Lilacs, Scielo, Cochrane, and Scopus databases including studies published in English between 1950 and March 2012. Cross-sectional, cohort, case control, and survey studies that assessed body posture in TMD patients were selected. Two reviewers performed each step independently. A methodological checklist was used to evaluate the quality of the selected articles.

**RESULTS:**

Twenty studies were analyzed for their methodological quality. Only one study was classified as a moderate quality study and two were classified as strong quality studies. Among all studies considered, only 12 included craniocervical postural assessment, 2 included assessment of craniocervical and shoulder postures,, and 6 included global assessment of body posture.

**CONCLUSION:**

There is strong evidence of craniocervical postural changes in myogenous TMD, moderate evidence of cervical postural misalignment in arthrogenous TMD, and no evidence of absence of craniocervical postural misalignment in mixed TMD patients or of global body postural misalignment in patients with TMD. It is important to note the poor methodological quality of the studies, particularly those regarding global body postural misalignment in TMD patients.

## Introduction

Temporomandibular Disorder (TMD) is a set of disorders characterized by signs and symptoms involving the temporomadibular joints and mastication muscles, as well as related structures^1^. There is evidence that its etiology is multifactorial and include psychological, biomechanical, and neurophysiological factors^2^^-^ 4.

The association between body postural changes and TMD has been widely discussed in the literature^5^^-^ 19. It is believed that in biomechanical terms, changes in head posture may be associated with the development and/or perpetuation of TMD^20^. Several studies over the last decades have reported the Forward Head Position (FHP) in patients with TMD^6^^,^ 12 ^,^ 20 ^,^ 21, however, these changes have not been verified in many other studies^5^^,^ 8 ^,^ 11 ^,^ 22.

Craniocervical posture is only one of the body segments that must be considered for postural assessment, specifically because adjacent postural compensations are expected in other segments considering that muscle chains are interconnected^23^^,^ 24.

Three systematic reviews regarding the theme were found in the literature^20^^,^ 25 ^,^ 26, however, the reviews by Olivo et al.^20^ and Rocha et al.^26^ only considered studies related to craniocervical posture and TMD, and the review by Perinetti and Contardo^25^ did not include studies on craniocervical posture. Moreover, this review^25^ classified, in the same list, studies regarding stabilometry (i.e. postural balance assessment) and static posture. Therefore, there was no systematic review available in the present literature involving body postural alterations (either segmentary or global) in individuals with TMD. Given the great interest in the theme and the poor methodological quality of the studies about body postural misalignment and the postural assessment methods employed in these studies^20^^,^ 25, it was important to carry out a study that analyzed real evidence of associations between static postural changes and TMD in order to guide better controlled studies in the future.

The confirmation of the evidence of the association between craniocervical or body postural misalignment and TMD may help to determine the predisposing and/or perpetuating factors in the development of TMD and guide new and well designed research to confirm this association. Moreover, some studies have demonstrated the relief of TMD symptoms after treatment involving postural reeducation^27^^,^ 28.

It was expected that the findings of this systematic review would demonstrate whether the evidence available was sufficient to indicate an association between body postural misalignment and TMD and/or subtypes. Thus, the aim of this study was to review the literature available on the main databases (i.e. PubMed/Medline, Embase, Lilacs, Scielo, Cochrane, and Scopus) about body postural misalignment in patients with TMD and subtypes.

## Method

### Data sources

In order to find studies examining the relationship between static body posture and TMD, bibliographical surveys were performed in the following databases: PubMed/Medline, Embase, Lilacs, Scielo, Cochrane, and Scopus. PRISMA^29^ (Preferred Reporting Items for Systematic reviews and Meta-Analyses) guidelines were followed.

The search comprised only studies in English published between 1950 and March 2012. The search terms were:

1) temporomandibular disorders
2) myofascial pain
3) stomatognathic system
4) craniofacial disorders

AND

1) body posture
2) head posture
3) body posture assessment
4) posture

Searches were performed by the same researcher. The limits of databases were selected when the option was available. In the Embase and Pubmed databases, the limits followed were: Published: 1966 to March 2012, quick limits: humans, only in English, article in press.

### Eligibility criteria

**Types of Studies**. i) cohort/case-control studies; and ii) cross-sectional and survey studies. Publications such as case reports, case series, reviews, and opinion articles were excluded. As the main objective of this study was to verify the possible association between TMD and body postural changes, randomized controlled clinical trials were excluded, since these studies are used to verify the effectiveness of an intervention and, therefore, not adequate to verify relationships between variables.

**Participants**. Inclusion was restricted to studies using human participants who (i) were between 7 and 60 years of age; (ii) had been diagnosed with TMD; (iii) had not previously had TMJ surgery; (iv) had no history of trauma or fracture in the TMJ or craniomandibular system; and, (v) had no other serious comorbid conditions (e.g. cancer, rheumatic disease, neurological problems).

### **Types of Outcome Measures**. The following methods of body postural assessment were considered: body landmarks, visual inspection, pictures or radiographs.

### Data collection

The reviewers analyzed all studies initially selected by the title or abstract for the inclusion/exclusion criteria. The published studies had to provide enough information to meet the inclusion criteria and not be eliminated by the exclusion criteria. In order for studies to be evaluated at the next level (critical appraisal), the study had to meet all of the inclusion criteria. When the reviewers disagreed on whether a study met a criterion, rating forms (form containing the Critical Appraisal completed by each reviewer - Table 1) were compared, and the criterion was discussed until a consensus was reached.

**Table 1:** Critical appraisal form used to evaluate included studies. Based on the paper by Olivo et al.(20).

Criteria for review and methodological quality assessment				
1) Type of Study				
a) Randomized Clinical Trial and Random / Cohort	S			
b) Pre-experimental / Non-randomized Clinical Study	M			
c) Case Control/ Cross-Sectional	W			
2) Diagnostic Criteria/Patients Assessment				
a) RDC/TMD Diagnostic	4			
b) American Academy of Orofacial Pain (AAOP) Criteria/Image	3			
c) Another Tool – Questionnaire	2			
d) Complaint or report	1			
e) Description of the groups: Myogenous / Arthrogenous / Mixed	1			
S = 4/M = 3/W < 2				
3) Volunteer Agreement				
a) >80%	S			
b) 60 to 80%	M			
c) <60%	W			
d) Cannot answer	W			
4) Sample Size Calculation				
a) Appropriate / A priori effect size and power	S			
b) Small, justification provided	M			
c) Small and no justification provided	W			
5) Method				
a) Visual Inspection – live Prior training of examiners Intrarater reliability Interrater reliability Reproducibility / Error Analysis Validity / Sensitivity / Specificity Well described	1 1 1 1 1 1 1	0 0 0 0 0 0 0	NA NA NA NA NA NA NA	
b) Qualitative Photographic Analysis Prior training of examiners Intrarater reliability Interrater reliability Reproducibility / Error Analysis Validity / Sensitivity / Specificity Well described	1 1 1 1 1 1	0 0 0 0 0 0	NA NA NA NA NA NA	
c) Quantitative Photographic Analysis				
Prior training of examiners Intrarater reliability Interrater reliability Reproducibility / Error Analysis Validity / Sensitivity / Specificity Well described	1 1 1 1 1 1	0 0 0 0 0 0	NA NA NA NA NA NA	
d) Radiography/Cephalometry Prior training of examiners Intrarater reliability Interrater reliability Reproducibility / Error Analysis Validity / Sensitivity / Specificity Well described	1 1 1 1 1 1 1	0 0 0 0 0 0 0	NA NA NA NA NA NA NA	

**Criteria for review and methodological quality assessment**				
For each item: S= 5 to 7 points/M = 4 to 3/W <2 NOTE: If an item was classified as NA (not applicable), it shoud be classified as follows: 0 to 33% of the items classified as NA = W/34 to 66% = M/ 67 to 100% = S				
6) Blinding				
Patients	1			Na
Examiner of the experiment	1	0		Na
Examiner the measure	1	0		Na
S= 2 or 3/ M = 1/ W = 0				
7) External validity				
Internal validity	1		0	
Good experimental design / selection bias				
Good control of confounding factors				
Appropriate statistical and sample calculation				
Consistency in results (validity / reliability / sensitivity)				
(1 point only if the paper achieve all items described)				
				
The results have clinical relevance	1		0	
Patients are representative of the population / where screened / age / comorbidities / severity	1		0	
Observed aspects were clarified in the conclusion and discussion	1		0	
S= 4 or 3/M = 2/W= 1 or 0				
8) Adequate statistical analysis				
a) Appropriate /suitable statistical tests	1		0	
b) Precision (P value described)	1		0	
c) Confidence Interval	1		0	
S :2/M: 1/W: 0				

S=Strong; M=Moderate; W=Weak; NA: Not applicable.

As recommended by PRISMA^29^, the studies were selected by the title, abstract, and full text. Two independent reviewers screened the abstracts of the publications found in the databases.

### Quality evaluation

In order to document the internal and external validity of the studies, a modified quality evaluation instrument was applied^20^^,^ 30. This tool considered: 1- study design, 2- control of confounding variables, 3- subjects' agreement to participate, 4- sample size calculation, 5- validity/reliability of outcomes measurements, 6- blinding, 7- external validity, and 8 - statistical analysis (Table 1). Two independent reviewers evaluated the studies based on specific determined criteria. If there was inadequate information in the published papers to allow evaluation of the criteria, the authors of the studies were contacted to clarify study design and specific characteristics of the study. If the authors did not reply, the studies were evaluated with the information available.

Each evaluated study item was then given a grade of strong (S), moderate (M) or weak (W) in each category. The rating system was based on a similar procedure^20^^,^ 31. Critical appraisal was completed independently by the two reviewers, and their results were compared. Data were extracted from each article without blinding of the authors. Finally, every study was graded depending on the following criteria (Table 1):

- STRONG - Strong for items: 2, 4, 5, 6, 7, and 8 *or* Moderate or Strong for items 1 and 3;
- MODERATE - Moderate for the following items: 2, 4, 5, 6, 7, and 8 and Weak or Moderate for items 1 and 3;
- WEAK - Weak for at least one of the items: 2, 4, 5, 6, 7, and 8.

### Statistical analysis

The kappa coefficient test was used to verify the agreement between both reviewers before the consensus stage in the analysis of studies. Results were obtained using the weighted kappa coefficient and analyzed using SPSS version 17, and the agreement was classified as follows: K<0.20 (poor), 0.21 to 0.40 (weak), 0.41 to 0.60 (moderate), 0.61 to 0.80 (good), 0.81 to 1.0 (excellent).

## Results

The selection included 1067 studies (271 in Pubmed, 3 in Scielo, 703 in Scopus, 33 in Lilacs, and 57 in Embase) considering duplicates/triplicates. After the removal of duplicates among different databases, 393 studies remained. After comparison for the existence of duplicates in the same database, 348 studies remained. The studies were screened again by verifying the title, and only 36 studies were selected.

Nevertheless, 16 studies were initially excluded after the abstract analysis based on the following inclusion and exclusion criteria : i) studies involving therapeutic intervention^28^^,^ 32 ^-^ 35; ii) sample eligibility criteria were not met (patients with TMD)^35^^-^ 38; iii) studies involving static balance assessment (stabilometry) or not involving static postural assessment^39^^-^ 41; and iv) non-experimental studies (i.e. letters to the editor, narrative literature reviews, pilot studies)^42^^-^ 45.

After analysis of the abstracts, all 20 studies were read once in full and five studies were excluded adopting the criteria previously defined. The studies were excluded because they consisted of: i) non-experimental studies^46^^,^ 47; ii) a study involving therapeutic intervention^27^; iii) a study involving static postural assessment^48^; and 4) a study with inappropriate sample eligibility criteria^49^.

**Figure 1:**
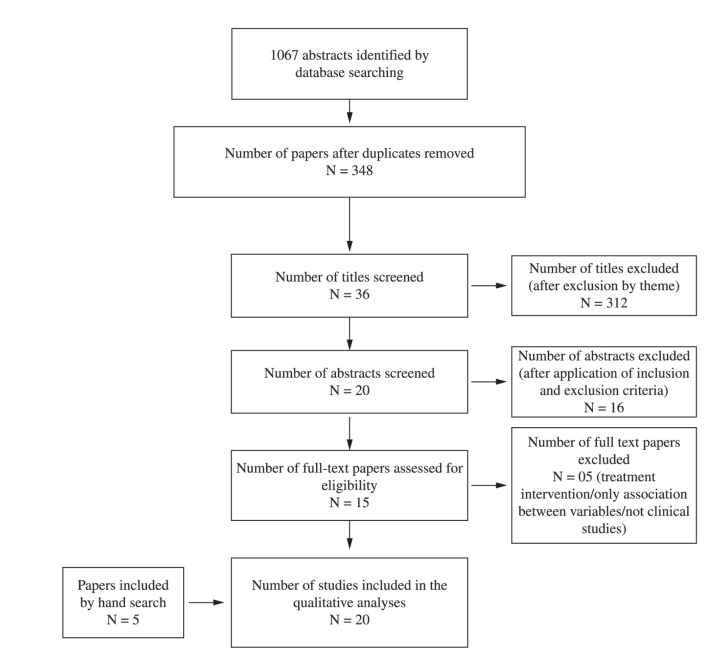
Flow diagram through the different phases of the systematic review as recommended by the PRISMA statement(30).

At the end of the process, through the selection by full text, a total of 15 studies were considered^5^^-^ 19. Later, 5 more studies were included through manual search^21^^,^ 22 ^,^ 50 ^-^ 52. Therefore, 20 studies in total were reviewed in the present study. All stages of this process are described in Figure 1.

The agreement between both reviewers for the final classification of the 20 studies obtained Interrater Kappa of 0.90 (Confidence Interval 95%: 0.73 1), demonstrating an excellent level of agreement between them.

### Quality criteria score

Considering the criteria for assessment of methodological quality, only three studies were classified as moderate^51^ or strong^19^^,^ 21. The main methodological problems observed were: 1) absence of description regarding sample size calculation^5^^-^ 18 ^,^ 49 ^,^ 50 (n=15 studies); 2) absence of reliability description of measures or validity of the method employed^5^^,^ 6 ^,^ 12 ^,^ 14 ^,^ 17 ^,^ 18 ^,^ 50 (n=7 studies); 3) absence of blinding of the examiners^6^^,^ 7 ^,^ 10 ^-^ 12 ^,^ 14 ^,^ 17 ^,^ 50 ^,^ 53 (n=9 studies); and, 4) non-compliance with criteria for internal and external validity^6^^,^ 7 ^,^ 10 ^,^ 11 ^,^ 13 ^-^ 15 ^,^ 18 ^,^ 52 (n=9 studies). Moreover, the randomization procedure for sample selection, which was observed in only six studies^5^^,^ 13 ^,^ 14 ^,^ 16 ^,^ 22, was still a significant bias that hindered the quality of the studies found in the literature^20^ (Table 2).

**Table 2:** Methodological scoring of the articles included in the review.

	Items / Score*								
Studies	1	2	3	4	5	6	7	8	Rating
**Craniocervical posture**									
Braun^6^	W	W	W	W	W	W	W	S	WEAK
Hackney et al.^11^	W	S	W	W	W	W	W	S	WEAK
Lee et al.^50^	W	W	W	W	W	M	S	S	WEAK
Evcik and Aksoy^12^	W	S	W	W	W	W	M	S	WEAK
Sonnensen et al.^10^	W	W	S	W	S	W	W	S	WEAK
Visscher et al.^8^	W	M	S	W	S	S	S	S	WEAK
D’Attilio et al.^51^	W	S	S	M	M	S	M	S	MODERATE
Munhoz et al.^13^	W	S	S	W	W	M	W	S	WEAK
Ioi et al.^52^	W	S	S	S	M	W	W	S	WEAK
Iunes et al.^22^	W	S	S	W	M	M	M	M	WEAK
Matheus et al.^15^	W	S	S	W	S	S	W	S	WEAK
De Farias Neto et al.^18^	W	S	S	W	W	S	W	S	WEAK
Armijo-Olivo et al.^19^	W	S	S	S	S	S	S	S	STRONG
Armijo-Olivo et al.^21^	W	S	S	S	S	S	S	S	STRONG
**Global Body posture**									
Darlow et al.^5^	W	W	W	W	W	M	M	S	WEAK
Zonnernberg et al.^7^	W	S	S	M	M	W	W	M	WEAK
Nicolakis et al.^9^	W	W	W	W	M	M	M	S	WEAK
Munhoz et al.^14^	W	S	S	W	W	W	W	S	WEAK
Munhoz et al.^16^	W	W	S	W	M	S	M	S	WEAK
Saito et al.^17^	W	S	S	W	W	W	M	M	WEAK
									
	W = 20	W= 6	W = 6	W = 15	W = 9	W = 8	W = 9	W = 0	
Total Score	M = 0	M = 1	M = 1	M = 2	M = 6	M = 5	M = 7	M = 3	
	S = 0	S = 13	S = 13	S = 3	S = 5	S = 7	S = 4	S = 17	

S=Strong; M=Moderate; W=Weak

*1- Types of studies; 2 - Diagnostic criteria; 3 - Volunteer agreement; 4 - Sample size; 5 - Method; 6 - Examiner blinding; 7 - External validity; 8 - Statistical analyses.

### Type of studies

Of all 20 studies considered, 12 studies were classified as case-control^5^^,^ 7 ^,^ 9 ^,^ 11 ^-^ 14 ^,^ 16 ^,^ 17 ^,^ 22 ^,^ 50 ^,^ 52 and eight were classified as cross-sectional^6^^,^ 8 ^,^ 10 ^,^ 15 ^,^ 18 ^,^ 19 ^,^ 21 ^,^ 51. Only three studies used random sampling in the process of group selection^5^^,^ 14 ^,^ 16 (Tables 3 and 4).

**Table 3:** Characteristics of the studies considered regarding temporomandibular disorders (TMD) and craniocervical posture.

Studies	Sample Size	Method used to assess posture	Criteria used for assessment/ diagnosis TMD	Results	Strengths and weaknesses
**Photographic metqhodaa**					
Braun^6^ – 1991 Postural differences between asymptomatic men and women and craniofacial pain patients Final Rating: WEAK Type of study: Cross-sectional study	N=49, unpaired Case Group: 9F Control Group: 40 (20F e 20M) - Case Group F: 38.11 (SD=6.95)years - Control Group: F: 28.4 (SD=9.29) years M: 29 (SD=4.39) years - sample size calculation – not mentioned - randomization to sample selection – not mentioned - Patients with mixed TMD attended at an orofacial pain clinic	- Photograph (sitting) + quantitative analysis - Forward Head Position (FHP) - Reliability of measurement – not mentioned - blinding of the examiner and previous training – not mentioned	Established criteria – not used	Greater angular shoulder extension in the symptomatic group Lower angle of FHP in the symptomatic group	• WEAKNESSES: - postural assessment training – not mentioned - blinding of the examiner - sample size calculation – not mentioned - Established criteria to TMD diagnosis – not mentioned • STRENGTHS: - suitable statistics - procedures well described
Hackney et al.^11^ *-* 1993 Relationship between forward head posture and diagnosed internal derangement of the temporomandibular joint Final Rating: WEAK Type of study: Case-control	N=44, paired - Case Group: 22 F: 19/M: 3 Mean age 38.6 years - Control Group: 22 F: 19/M: 3 Mean age 35.4 years - sample size calculation – not mentioned - randomization to sample selection – not mentioned - Paients with TMD arthrogenic – selected from a TMD clinic	- Photograph in sitting and standing posture - quantitative analysis - Register and analysis performed ​​by the same examiner - Report previous examiner training - blinding of the examiner – not mentioned - Report consistency between – without use of suitable statistics	Established criteria – not used Clinical examination confirmed by MRI	Without differences between groups	• WEAKNESSES: - sample size is not justified - examiners blinding – not mentioned - reliability – not mentioned - Established diagnostic criteria – not used • STRENGTHS: - paired sample - adequate statistic - diagnosis confirmed by imaging
Lee et al.^50^ - 1995 The relationship between forward head posture and temporomandibular disorders. Final Rating: WEAK Type of study: Case-control	- N: 66, paired (age and gender) - Case Group: 33 F: 30/M: 3 Mean age: 31.4 (SD=10.1) years - Control group: 33 F: 19 M: 3 Mean age: not reported - sample size calculation – not mentioned - randomization to sample selection – not mentioned - Patients with mixed TMD selected from an orofacial pain center at the Kentucky University	- Craniocervical and shoulder photographs - reliability of the measure and method – not mentioned - blinding of the examiner – not mentioned	Established criteria – not used	- Forward Head Position angle lower in patient group - Protrusion head higher in patients with TMD	• WEAKNESSES: - calibration of raters – not mentioned - method reliability – not mentioned - examiners blinding – not mentioned - Established diagnostic criteria – not used - sample size is not justified • STRENGTHS: - paired grouvps - procedures well described - adequate statistic - blinding of patient
Evcik and Aksoy^12^ - 2000 Correlation of TMJ pathologies, neck pain and postural differences Final Rating: WEAK Type of study: Case-control	N: 38, unpaired. - Case Group: 18 F: 15 - 30.4 (7.6) years M: 3 - 30.4 (8.7) years Mean age: 28.5 (SD=12.93) - Control Group: 20 F: 15 M: 5 Mean age: 29.7 (SD=9.76) - sample size calculation – not mentioned - randomization to sample selection – not mentioned - Patients with arthrogenous TMD	- Posture photographs and quantitative analysis (lateral photograph) - Information about the examiners (blinding, training or reliability) – not mentioned	*-* Established criteria – not mentioned TMD detailed clinical examination + TMJ MRI	Lower FHP angle in TMD Greater shoulder protrusion in TMD	• WEAKNESSES: - unpaired sample - sample size is not justified - examiners blinding – not mentioned - reliability – not reported • STRENGTHS: - adequate statistic - confirmation of diagnostic by imaging
Armijo-Olivo et al.^21^ - 2011 Head and cervical posture in patients with temporomandibular disorders Final Rating: STRONG Type of study: Cross-sectional study	N: 172 - Myogenous TMD Group: F/M: 55, mean age: 31.91 (SD=9.15) years - Mixed TMD Group: F/M: 49, mean age: 30.88 (SD=8.19) years - Control Group: F/M: 50, mean age: 28.28 (SD=7.26) years - Sample size calculation - randomization of the selected sample was not mentioned - Patients with myogenous and mixed TMD selected from a orofacial pain clinic at the University of Alberta	- Lateral photographs of posture - Reliability of measurement ICC: 0.99 - Training of examiner - Blinding of the examiners	- RDC/TMD	- Difference for the eye-tragus-horizontal angle for myogenous TMD patients compared to controls (i.e. greater head extension)	• WEAKNESSES: - randomization of the sample – not mentioned - Validity of the method, not demonstrated • STRENGTHS: - adequate statistic - sample size is justified - procedures well described - reliability of the measurements
Armijo-Olivo et al.^19^ - 2011 Clinical relevance vs. statistical significance: Using neck outcomes in patients with temporomandibular disorders as an example Final Rating: STRONG Type of study: Cross-sectional study	N=154 - Case Group: with myogenous TMD - F/M: 56 with mixed TMD – F/M: 48 - Control Group: F/M: 50 - Sample size calculation - randomization of the selected sample was not mentioned - Patients with myogenous and mixed TMD selected from an orofacial pain clinic at the University of Alberta	- Lateral photographs of posture - Reliability of measurement reported in a previous publication - Armijo-Olivo et al.^19^ (2011) - Report of previous training examiner - blinding of the examiners	- RDC/TMD	- Difference for the eye-tragus-horizontal angle in myogenous TMD patients compared to controls – head extension - The effect size was 0.48 (the authors consider a statistical difference, but not clinical)	• WEAKNESSES: - Randomization of the sample - Validity of the method, but does not show it • STRENGTHS: - sample size is justified - procedures well described - reliability of the measurements - adequate statistics
**Radiographic method**					
Sonnesen et al.^10^ - 2001 Temporomandibular disorders in relation to craniofacial dimensions, head posture and bite force in children selected for orthodontic treatment. Final Rating: WEAK Type of study: Cross-sectional study	N: 96 children - 51 girls and 45 boys, between 7 and 13 years of age - sample size calculation – not mentioned - randomization to sample selection – not mentioned - Patients with mixed TMD - Children admitted for orthodontic treatment in a dental service	- Postural assessment by radiography - Cephalometric radiography - Excellent reliability of cephalometric tracings (ICC: 0.97 to 1.00) - blinding of the examiner – not mentioned	*-* It did not use established criteria - Good and excellent reliability assessment of TMD	Low and moderate correlation (r: 0.21 to 0.37) between cervical posture and craniocervical and pain on palpation of the masticatory muscles, neck and shoulders - Head extension in TMD	• WEAKNESSES: - Standardized criteria- not used - sample size is not justified - examiners blinding – not mentioned • STRENGTHS: - reliability and calibration of raters - procedure well described
D’Attilio et al.^51^ - 2004 Cervical lordosis angle measured on lateral cephalograms; findings in skeletal class II female subjects with and without TMD: a cross sectional study Final Rating: MODERATE	N=100; unpaired (but similar age range) - Case Group: F: 50; mean age 28.6 (SD=3.3) years - Control Group: F:50; mean age 29.3 (SD=3.2) years - sample size calculation – not mentioned - randomization to sample selection – not mentioned - Paients with TMD arthrogenous (disk displacement with and without pain )	- Cephalometric radiography - SE^2^ = Σ D^2^⁄ 2n (where, SE is the standard error, D is the difference between duplicated measurements, and “n” is the number of duplicated measurements) - Blinding of the examiner	- TMD: clinical assessment + MRI + X-ray - The same blinded examiner	Lower Cervical lordosis angle (CVT/EVT) – for TMD compared to control group	• WEAKNESSES: - sample size is not justified • STRENGTHS: - TMD assessed by image - reliability and error analysis - suitable statistics
Munhoz et al.^13^ *-* 2004 Radiographic evaluation of cervical spine of subjects with temporomandibular joint internal disorder Final Rating: WEAK Type of study: Case-control	N: 50 *-* Case Group: 30 F: 27 M: 3 Mean age: 22.9 (SD=5.3) years - Control Group: 20 F:14/M: 6 Mean age: 21.7 (SD=3.6) years - sample size calculation – not mentioned - randomization to sample selection – not mentioned - 3 blinded examiners - Patients with arthrogenous and mixed TMD Selected from a TMD clinic at the University of São Paulo	- Radiographic posture analysis + quantitative and qualitative analysis - Agreement between raters - Viikari-Juntura^56^ method - Blinding of the examiner	- TMD: interview + clinical assessment AAOP (to select) + Helkimo^57^ - image analysis – not used	- There was not difference between groups	• WEAKNESSES: - sample size is not justified - unpaired sample • STRENGTHS: - adequate statistics - blinded examiners - reliability - TMD case definition = AAOP
Ioi et al.^52^ - 2008 Relationship of TMJ osteoarthritis to head posture and dentofacial morphology Final Rating: WEAK Type of study: Case-control	N: 59, unpaired - Case Group: F: 34 (patients) mean age: 24.7 (SD=6.1) years - Control Group: F: 25 (university and employees) mean age: 23.6 (SD=1.3) anos - Sample size calculation - Randomization of the selected sample – not mentioned - Patients with arthrogenous TMD	- Radiographic posture analysis - Examiners were blinded – not mentioned - Dahlberg error method: lower than 0.58 mm and 0.61 degrees Dahlberg method error: SE^2^= SΣd^2^/2n (where, SE = Stantard error, d = difference between repeated measurements and n = the number of records)	Muir and Goss^58^ arthrogenous TMD criteria (1990) - Radiography	- Craniocervical angles greater in TMD	• WEAKNESSES: - unpaired sample - Examiners blinding – not mentioned • STRENGTHS: - sample size is justified - adequate statistic - Error analysis of measurements - confirmation of diagnostic by imaging
Matheus et al.^15^ - 2009 The relationship between temporomandibular dysfunction and head and cervical posture Final Rating: WEAK Type of study: Cross-sectional study	N: 60 F: 47/M: 13 Mean age: 34.2 years Case Group: 39 Control Group: 21 - sample size calculation – not mentioned - randomization to sample selection – not mentioned - Patients with arthrogeneous and mixed TMD	- Cephalometric analysis of radiographic craniocervical posture - measurement reproducibility - Blinding of the examiner	*-* RDC/TMD + MRI examination - Experts and blinded examiners to MRI	Disk displacement and neck posture – no association	• WEAKNESSES: - sample size is not justified - comparisons among small groups • STRENGTHS: - procedures well described - experts and blinded examiners - reproducibility of measurement - adequate statistics - RDC/TMD used - confirmation of diagnostic by imaging
de Farias Neto et al.^18^ - 2010 Radiographic measurement of the cervical spine in patients with temporomandibular disorders Final Rating: WEAK Type of study: Cross-sectional study	N=56 - Case Group (12): M: 5, mean age 24 (SD=3.1) years F: 7, mean age 21.4 (SD=4.4) years - Control Group (11): M: 4, mean age 19 (SD=0.8) years F: 7, mean age 20.6 (SD=3) years - sample size calculation – not mentioned - randomization to sample selection – not mentioned - Patients with mixed TMD Research subjects in treatment at a clinic of orofacial pain	- Lateral radiographs - reliability of the measures – not mentioned - blinding of the examiner	- RDC/TMD	- Differences in atlas plane angle from the horizontal and anterior translation Greater flexion of the first cervical vertebra, associated with cervical hyperlordosis in TMD	• WEAKNESSES: - reliability measures – not mentioned - small sample size - sample size calculation – not mentioned
**Photographic and radiographic method**					
Visscher et al.^8^ - 2002 Is there relationship between head posture and craniomandibular pain? Final Rating: WEAK Type of study: Cross-sectional study	N=250 Case group: 138 However, only 130 were subjected to postural analysis (8 patients had lost points in radiographic analysis) TMD Group: 16 Cervical dysfunction Group: 10 Mixed Group: 59 Control Group: 45 3 Cases Groups: Temporomandibular Disorders (TMD) Group Cervical Spine Disorders (CSD) Group TMD and CSD Group (both conditions together) - sample size calculation – not mentioned - randomization to sample selection – not mentioned - Patients with arthrogenous, myogenous and mixed TMD consecutively selected from a dental clinic	- Photography in sitting and standing + head/cervical X-ray - Reliability of photographic method- ICC: 0.96 - Blinding of the examiner - Experts, calibrated and blinded examiners	*-* Established criteria – not used	No differences for head posture measurements between the groups	• WEAKNESSES: - unpaired sample - standardized criteria to diagnosis – not used - despite being large, the sample was subdivided into 4 groups • STRENGTHS: - adequate statistic - procedures well described - experts examiners, calibrated and blinded – reliability reported
Iunes et al.^22^ - 2009 Craniocervical postural analysis in patients with TMD Final Rating: WEAK Type of study: Case-control	N= 90 women, paired - Group 1: F: 30 (myofascial disorders ) mean age: 29.13 (SD=11.45) years - Group 2: F: 30 (mixed TMD) mean age: 28.13 (SD=9.42) years - Control Group: F: 30 (asymptomatic) mean age: 26.17 (SD=9.18) years - sample size calculation – not mentioned - randomization to sample selection – not mentioned - Patients with myogenous and mixed TMD	- Radiography and photograph to perform posture analysis - quantitative and qualitative analysis - Blinding of the examiners - Reliability analysis of radiographic: ICC between 0.76 and 0.99	*-* RDC/TMD - Examiner training – not mentioned	- PHOTOGRAPH: no difference - RADIOGRAPH: no difference - VISUAL ANALYSIS: no difference	• WEAKNESSES: - sample size is not justified, but suitable • STRENGTHS: - case definition: RDC/TMD - blinded and trained examiners - procedures well described

F: Female, M: Male; N: Sample Size; SD: Standard deviation; RDC/TMD: Research Diagnostic Criteria for Temporomandibular Disorders; MRI: Magnetic Resonance Image; AAOP: American Academy of Orofacial Pain; CVT/EVT: Cervical lordosis angle. The downward opening angle between the CVT and EVT line; CVT: A line through the tangent point of the superior, posterior extremity of the odontoid process of the second cervical vertebra and the most infero-posterior point on the body of the fourth cervical vertebra; EVT: A line through the most infero-posterior point on the body of the fourth cervical vertebra and the most inferoposterior point on the body of the sixth cervical vertebra; TMJ:Temporomandibular joint.

**Table 4:** Characteristics of the studies considered regarding TMD and global body posture.

Studies	Sample Size	Method used to assess posture	Criteria used for assessment/ diagnosis TMD	Results	Strengths and weaknesses
**Visual Inspection**					
Darlow et al.^5^ - 1987 The relationship of posture to miofascial pain dysfunction syndrome Final Rating: WEAK Type of study: Case-control	N=60, paired Case Group: 30 F: 23, mean age 35.8 years M: 7, mean age 38 years Control Group: 30 (23F & 7M) F: 23, mean age 29.3 years M: 7, mean age 35.3 years - sample size calculation – not mentioned - randomization of the selected sample was mentioned - Patients with myogeneous TMD assisted in a facial pain program at a hospital	- Visual inspection by Kendall et al.^59^ method - parameters graded on a scale 0-5 - Previous training of the examiner reported - reliability of measurement – not mentioned - blinding of the examiner – not mentioned	*-* Established diagnosis criteria – not used	- No differences between the groups	• WEAKNESSES: - sample size is not justified - TMD definition not established criteria - reliability of the measurement – not mentioned • STRENGTHS: - paired sample - adequate statistic - trained and blinded examiner
Nicolakis et al.^9^ - 2000 Relationship between craniomandibular disorders and poor posture Final Rating: WEAK Type of study: Case-control	N=50, paired (age and gender) - Case Group: 25 F: 20, mean age: 28.9 (SD=7.5) years M: 5 , mean age: 25.8 (SD=2.8) years - Control Group: 25 F: 20, mean age: 28.8 (SD=5) years M: 5, mean age: 26.4 (SD=1.5) years - Sample size calculation – not mentioned - randomization of the selected sample – not mentioned - Patients with mixed TMD selected consecutively at the Department of Dentistry and the control group from the University	- visual inspection by Kendall et al.^59^ method - Always the same trained examiner - Reproducibility and reliability of the measures tested in previous studies	*-* Established criteria – not used	- Greater number of postural changes for neck and trunk in the frontal and sagittal planes in the TMD	• WEAKNESSES: - sample size is not justified - Established Diagnostic criteria – not used • STRENGTHS: - paired sample - blinded examiner - reliability and reproducibility of the measure - adequate statistic
Saito et al.^17^ - 2009 Global body posture evaluation in patients with temporomandibular joint disorder. Final Rating: WEAK Type of study: Case-control	N: 26 woman - Control Group: F:16, mean age: 24.4 (SD=2.8) years - Case Group: F:10, mean age: 24.5 (SD=3) years - sample size calculation – not mentioned - randomization of the selected sample – not mentioned - Patients with arthrogenous TMD	- Visual inspection by Kendall et al.^59^ method - expert examiner - procedures for photographic record – poorly described - blinding of the examiner – not mentioned	- Interview + clinical assessment + image (X-ray)	Postural changes on the hip, thoracic curve flatted and increased lumbar lordosis in TMD Greater lateral flexion of the head in patients with TMD	• WEAKNESSES: - sample size is not justified - posture procedures not well described - reliability of the method – not mentioned - blinding of the examiners – not mentioned • STRENGTHS: - paired sample - discusses some limitations of the study - TMD diagnostic by imaging
**Photographic Method**					
Zonnenberg et al.^7^ - 1996 Body posture photographs as a diagnostic aid for musculoskeletal disorders related to TMD Final Rating: WEAK Type of study: Case-control	N=80, paired (age and gender) - Case Group: 40 F: 33, mean age: 30.4 (SD=7.6) years M: 7, mean age: 30.4 (SD=8.7) years - Control Group: 40 F: 32, mean age: 35.5 (SD=9.8) years M: 8, mean age: 30.4 (SD=8.7) years - Sample size calculation – not mentioned - randomization of the selected sample – not mentioned - Patients with mixed TMD	- Photographs of body posture (quantitative) - Good reliability of measurement (previous study) - blinding of the examiner	- Established criteria for diagnosis (AAOP)	- Greater tilt of the lines between the pupils and pelvis in TMD patients	• WEAKNESSES: - TMD not assessed in controls - sample size is not justified, but reasonable/moderate - blinding or training of examinrs – not mentioned - posture analysis only in frontal plane • STRENGTHS: - paired sample - TMD definition by AAOP - reliability of the measure (previous publication)
Munhoz et al.^14^ - 2005 Evaluation of body posture in individuals with internal temporomandibular joint derangement Final Rating: WEAK Type of study: Case-control	N=50, unpaired / college students *-* Case Group: 30 F: 27/M: 3 mean age: 21.7 (SD=3.6) years - Control Group: 20 F: 14/M: 6 mean age: 22.9 (SD=5.3) years - Sample size calculation – not mentioned - randomization of the selected sample - Patients with arthrogeneous and mixed TMD selected from a TMD clinic	- Photograph to assess posture - quantitative analysis - reliability of measurement and the method - blinding of the examiner – not mentioned	- TMD: interview + clinical assessment AAOP + Helkimo^57^	- No differences between groups	• WEAKNESSES: - sample size is not justified - unpaired sample - reliability of the method – not mentioned - training or blinding of examiners – not mentioned • STRENGTHS: - adequate statistic - AAOP criteria for TMD diagnosis
Munhoz and Marques^16^- 2009 Body posture evaluations in subjects with internal temporomandibular joint derangement Final Rating: WEAK Type of study: Case-control	N=50, paired - Case Group: 30 F: 27/M: 3 mean age: 21.7 (SD=3.6) years - Control Group: 20 F: 16/M: 6 Mean age: 22.9 (SD=5.3) years - sample size calculation – not mentioned - randomization of the selected sample - Patients with arthrogeneous and mixed TMD selected from a TMD clinic	- Photograph records used to perform qualitative posture analysis - blinding of the examiners - Interrater agreement: low reliability (below 0.52)	TMD: questionnaire + Helkimo	- TMD patients presented - lifting shoulders and on hip posture deviations	• WEAKNESSES: - sample size is not justified *-* Established diagnosis criteria – not used - low interrater agreement • STRENGTHS: - randomization of the sample - suitable statistics - blinded examiners

F: female; M: male; N: sample size; SD: standard deviation; AAOP: American Academy of Orofacial Pain.

### TMD assessment/Diagnosis criteria

Seven studies used diagnosis criteria that are not well established in the literature^5^^,^ 6 ^,^ 9 ^,^ 10 ^,^ 12 ^,^ 16 ^,^ 50. Image analysis were employed in four studies^11^^,^ 17 ^,^ 51 ^,^ 52, the criterion of the American Academy of Orofacial Pain (AAOP) in three studies^7^^,^ 13 ^,^ 14, and the Research Diagnostic Criteria for Temporomandibular Disorders (RDC/TMD)^3^^,^ 4 in five studies^15^^,^ 18 ^,^ 19 ^,^ 21 ^,^ 22 (Tables 3 and 4).

### Segmental or global body postural assessment

Of all studies included in this review, six used body postural assessment^5^^,^ 7 ^,^ 9 ^,^ 14 ^,^ 16 ^,^ 17, five assessed only craniocervical posture and shoulders^6^^,^ 12 ^,^ 19 ^,^ 21 ^,^ 23, and all others assessed only craniocervical and/or cervical posture^8^^,^ 10 ^,^ 11 ^,^ 13 ^,^ 15 ^,^ 18 ^,^ 50 ^-^ 52.

### Sample size, posture method assessment, and examiner blinding

Sample size was calculated in only three studies^19^^,^ 21 ^,^ 49 (Table 2). Of the studies that analyzed only craniocervical posture, six studies described the use of assessment by radiographic analyses^10^^,^ 13 ^,^ 15 ^,^ 18 ^,^ 49 ^,^ 51, six studies used the photographic method^6^^,^ 11 ^,^ 12 ^,^ 19 ^,^ 21 ^,^ 50, and two described the use of the both radiographic and photographic methods^8^^,^ 22 (Tables 3 and 4).

Only six studies assessed global body posture^5^^,^ 7 ^,^ 9 ^,^ 14 ^,^ 16 ^,^ 17. Five used the visual inspection method^5^^,^ 9 ^,^ 14 ^,^ 16 ^,^ 17, one used the quantified photographic method^7^, and one used the photographic method with qualitative analysis^14^ (Table 4). Eleven studies described examiner blinding to assess body or craniocervical posture^5^^,^ 8 ^,^ 9 ^,^ 13 ^,^ 15 ^,^ 16 ^,^ 18 ^,^ 19 ^,^ 21 ^,^ 22 ^,^ 51 (Tables 3 and 4).

Considering the reliability of the body posture measures, seven studies did not provide this information accurately^5^^,^ 6 ^,^ 11 ^,^ 12 ^,^ 17 ^,^ 18 ^,^ 50, 11 reported good levels of reliability among the repeated measures^7^^-^ 10 ^,^ 13 ^,^ 15 ^,^ 19 ^,^ 21 ^,^ 22 ^,^ 51 ^,^ 52, and one study reported poor reliability^16^ (Tables 3 and 4).

Only one of the studies included in this review mentioned the validity of the measures employed for postural assessment^22^, however the reference that certified the method validity was probably incorrect^54^. The authors did not answer the e-mail to clarify this possible error.

Of the six studies using global body posture, the standardization for posture analysis and analysis method was appropriately described in three studies^7^^,^ 14 ^,^ 16. The photogrammetry method was used by two studies^7^^,^ 14 and a previously described method combining photographic and visual inspection was used in one study^16^ (Table 4).

### Postural changes in TMD

Body posture changes in the group of patients with TMD in relation to a control group was verified in 13 studies^6^^,^ 7 ^,^ 9 ^,^ 10 ^,^ 12 ^,^ 16 ^-^ 19 ^,^ 21 ^,^ 50 ^-^ 52 (Tables 3 and 4). Among the studies that assessed craniocervical posture (n=20), 10 studies reported misalignment in the TMD group^6^^,^ 7 ^,^ 9 ^,^ 10 ^,^ 12 ^,^ 17 ^,^ 19 ^,^ 21 ^,^ 50 ^,^ 52. Three studies verified alterations in FHP angle^6^^,^ 12 ^,^ 50 and two studies^19^^,^ 21 used another angle measurement (eye-tragus-horizontal angle). In all of the studies, head protrusion or extension was observed. Considering the five studies that performed specific measurements of the cervical spine^8^^,^ 14 ^,^ 18 ^,^ 22 ^,^ 51, changes of this segment were observed in two studies^18^^,^ 51. Upper cervical spine flexion and hyperlordosis were reported by De Farias Neto et al.^18^ and cervical spine straightening by D'Áttilio et al.^51^ (Tables 3 and 4).

Of the studies that verified shoulder postural changes^5^^-^ 7 ^,^ 9 ^,^ 12 ^,^ 14 ^,^ 16, four studies verified posture changes in this segment in the TMD group^6^^,^ 9 ^,^ 12 ^,^ 16. The misalignments were: greater shoulder extension^6^, assymetrical shoulders and abducted scapula^9^, shoulder protrusion^12^, and elevated shoulder^16^ (Tables 3 and 4).

Among the six studies that assessed pelvic posture, four studies^7^^,^ 9 ^,^ 16 ^,^ 17 verified pelvic misalignments in the frontal plane^7^, iliac crest^9^, muscle chain^16^, and posterior rotation^17^ (Tables 3 and 4). Spinal misalignments were identified by two of the five studies that included this topic in the postural assessment^5^^,^ 9 ^,^ 14 ^,^ 16 ^,^ 17: greater thoracic kyphosis and lumbar hyperlordosis^9^ and kyphosis straightening and lumbar hyperlordosis^17^ (Table 4). However, of the studies that were classified as moderate or strong quality, Armijo-Olivo et al.^19^^,^ 21 reported greater head extension and D'Áttilio et al.^51^ observed cervical spine straightening.

### Postural changes in TMD subtypes

Of the five studies that included a group of patients with myogenous TMD^5^^,^ 8 ^,^ 19 ^,^ 21 ^,^ 22, two found body posture misalignments (head extension) in the TMD group in relation to the control group or mixed TMD group^19^^,^ 21. Both studies were classified as strong according to the adopted quality criteria applied (Tables 3 and 4).

Concerning arthrogenous TMD, four studies verified body posture changes in the TMD group in relation to the control group or another TMD group^12^^,^ 17 ^,^ 51 ^,^ 52, and three did not report craniocervical postural changes^8^^,^ 11 ^,^ 15. Only the study by D'Áttilio et al.^51^ was classified as moderate quality. The authors reported cervical spine straightening (Tables 3 and 4).

Among the studies that included a group of mixed TMD patients in relation to a control group or another TMD group^6^^-^ 10 ^,^ 16 ^,^ 18 ^,^ 19 ^,^ 21 ^,^ 22 ^,^ 50, seven reported body posture alterations^6^^,^ 7 ^,^ 9 ^,^ 10 ^,^ 16 ^,^ 18 ^,^ 50. Only two studies^19^^,^ 21 were classified as strong quality and they did not report body posture alterations for the mixed TMD group (Tables 3 and 4), however in both studies this group had to have a diagnosis of myiogenous TMD according to the RDC/TMD but not a diagnosis of arthrogenous TMD according to these criteria, only signs and symptoms.

## Discussion

The purpose of this systematic review was to identify the level of scientific evidence for the association between TMD and body and/or craniocervical posture misalignment. The quality criteria adopted for review of the studies have been described in previous studies^20^ and the agreement between the reviewers for the methodological classification of the studies was high (kappa: 0.91), demonstrating that the review process was considered reliable.

This systematic review considered global body posture misalignment. Regarding the three systematic reviews on the subject, two of them considered craniocervical posture only^20^^,^ 26 and the other presented records of static posture that were analyzed together with records of balance - static posturography^25^. Moreover, these authors^25^ disregard studies about craniocervical posture. Postural assessments aimed at finding postural deviations are routinely made by physical therapists to analyze body segments in the static position and do not include the assessment of oscillations that must be considered as balance assessment.

A significant number of the studies found in the literature and included in this review (n=14) considered only the assessment of the head segment^6^^,^ 8 ^,^ 10 ^-^ 13 ^,^ 15 ^,^ 18 ^,^ 19 ^,^ 21 ^,^ 22 ^,^ 50 ^-^ 52. This aspect is probably related to the fact that it is easier to perform the procedure in the craniocervical segment, since the individual does not need to be evaluated in bathing clothes, and moreover because the radiographic procedure commonly employed in dentistry only considers the head and cervical spine, and it does not enable the analysis of global body posture. On the other hand, this aspect disregards posture assessment as a whole and it is possible that head changes are related to distal changes, since the connection between the muscles through the muscular chains would facilitate the emergence of postural compensation in other body segments^23^.

### Main findings and TMD subtypes

This review demonstrated that there is evidence for craniocervical postural change (i.e. head extension) in patients with myogenous TMD in relation to controls. Of the five studies that included a group of patients with myogenous TMD^5^^,^ 8 ^,^ 19 ^,^ 21 ^,^ 22, two studies were classified as strong according to the quality criteria employed and verified only craniocervical posture changes in TMD in relation to a control group or a mixed TMD group^19^^,^ 21.

Considering body posture misalignment in arthrogenous TMD, only the study of D'Attilio et al.^51^ was classified as moderate according to the criterion quality adopted. Therefore, it was observed that there was moderate evidence and risk of bias for the presence of cervical posture misalignment (i.e.cervical spine straightening) in patients with arthrogenous TMD, diagnosed by MRI, in relation to a control group.

Considering studies involving patients with mixed TMD, only two studies^19^^,^ 21 obtained a strong classification according to the quality criteria adopted and they did not report body postural misalignment for the mixed TMD group. One of the reasons for the absence of evidence of body postural misalignment in mixed TMD patients compared to myogenous and arthrogenous patients could be related to the sample selection adopted^19^^,^ 21. The patients should have a diagnosis of myogenous TMD according to the RDC/TMD associated with signs and symptoms of arthrogenic TMD. In this way, all of the patients must have a diagnosis of myogenous TMD, but not of arthrogenous TMD. It is possible that the "mixed TMD group" could not fill the criteria for an arthrogenous TMD diagnosis, since signs and symptoms of arthrogenous complaints have commonly been observed in the population^55^. Hence, there is no evidence that patients with mixed TMD (i.e. with myogenous TMD diagnosis and signs and symptoms of arthrogenous TMD) did not have body or craniocervical misalignment in relation to individuals without TMD or myogenous TMD.

D'Atillio et al.^51^ demonstrated cervical spine straightening in arthrogenous TMD patients and received a moderate evidence level classification. However, D'Atillio et al.^51^ used radiographic analysis to assess cervical spine misalignment and Armijo-Olivo et al.^19^^,^ 21 verified only head and cervical/head posture. In this way, it is possible that in patients with arthrogenous TMD, cervical spine misalignment could be more common, and in patients with myogenous TMD disorders, head posture misalignment could be more common. It could explain the absence of body posture misalignment for mixed TMD group described by Armijo-Olivo et al.^19^^,^ 21.

However, all of these theories are speculative and the attention should focus on the need for future studies to include a large sample size, control the diagnostic criteria for mixed and arthrogenous groups, and consider not only photographic records but also radiographic procedures to analyze the cervical spine more specifically. Two studies assessed body posture by both photography and radiography^8^^,^ 22, however the major flaw of these papers was their limited sample size. Armijo-Olivo et al.^19^ described a minimum of 50 subjects (α=0.05, β=0.20, power=80%, and effect size of 0.5) to assess posture by photographic records.

Global body postural misalignment in the group of TMD patients was verified in four studies^7^^,^ 9 ^,^ 16 ^,^ 17. All studies obtained a weak classification. Aspects such as absence of blinding of the examiner^7^^,^ 17, failure in sample eligibility criterion^9^^,^ 16, and poorly described or undescribed reliability of the method^5^^,^ 16 ^,^ 17 were some of the characteristics that did not support the evidence of possible global body postural changes in arthrogenous, myogenous or mixed TMD groups in relation to a control group.

As contribution for future publications, the authors recommend effect size and power analysis, a more controlled design, appropriate description of reliability/validity of the measures (specifically for global body postural assessment), blinding of the examiners, random sampling, and, eligibility criteria of patients with control of subtypes of TMD according to well stablished criteria.

## Conclusion

The main contributions of the present review are the following: there is evidence and low risk of bias that patients with myogenous TMD have craniocervical postural misalignment. For the arthrogenous TMD group, moderate evidence for cervical spine alterations was observed. Moreover, there was no evidence in the literature for the absence of craniocervical posture misalignment in mixed TMD patients and for global body posture misalignment in TMD. The poor methodological quality of the studies considered in this revision, especifically for body postural misalignment could be the explanation for the weak evidence observed.
